# OxyHbMeter—a novel bedside medical device for monitoring cell-free hemoglobin in the cerebrospinal fluid—proof of principle

**DOI:** 10.3389/fmedt.2024.1274058

**Published:** 2024-04-11

**Authors:** Nikolaos Tachatos, Jan Folkard Willms, Michael Sebastian Gerlt, Kiran Kuruvithadam, Michael Hugelshofer, Kevin Akeret, Jeremy Deuel, Emanuela Keller, Marianne Schmid Daners

**Affiliations:** ^1^ Product Development Group Zurich, Department of Mechanical and Process Engineering, ETH Zurich, Zurich, Switzerland; ^2^ Neurointensive Care Unit, Department of Neurosurgery and Institute of Intensive Care Medicine, Clinical Neuroscience Center, University Hospital Zurich and University of Zurich, Zurich, Switzerland; ^3^ Biomedical Engineering Department, Lund University, Lund, Sweden; ^4^ Department of Neurosurgery, Clinical Neuroscience Center, University Hospital Zurich and University of Zurich, Zurich, Switzerland; ^5^ Department of Medical Oncology and Haematology Clinic, University Hospital Zurich and University of Zurich, Zurich, Switzerland; ^6^ Department of Mechanical and Process Engineering, Institute for Dynamic Systems and Control, ETH Zurich, Zurich, Switzerland

**Keywords:** delayed cerebral ischemia, aneurysmal subarachnoid hemorrhage, microfluidics, acoustic particle manipulation, cell separation, point of care device, centrifugation, spectrophotometry

## Abstract

Delayed cerebral ischemia (DCI) occurs in up to one third of patients suffering from aneurysmal subarachnoid hemorrhage (aSAH). Untreated, it leads to secondary cerebral infarctions and is frequently associated with death or severe disability. After aneurysm rupture, erythrocytes in the subarachnoid space lyse and liberate free hemoglobin (Hb), a key driver for the development of DCI. Hemoglobin in the cerebrospinal fluid (CSF-Hb) can be analyzed through a two-step procedure of centrifugation to exclude intact erythrocytes and subsequent spectrophotometric quantification. This analysis can only be done in specialized laboratories but not at the bedside in the intensive care unit. This limits the number of tests done, increases the variability of the results and restricts accuracy. Bedside measurements of CSF-Hb as a biomarker with a point of care diagnostic test system would allow for a continuous monitoring for the risk of DCI in the individual patient. In this study, a microfluidic chip was explored that allows to continuously separate blood particles from CSF or plasma based on acoustophoresis. An *in vitro* test bench was developed to test in-line measurements with the developed microfluidic chip and a spectrometer. The proof of principle for a continuous particle separation device has been established with diluted blood and CSF samples from animals and aSAH patients, respectively. Processing 1 mL of blood in our microfluidic device was achieved within around 70 min demonstrating only minor deviations from the gold standard centrifugation (7% average error of patient samples), while saving several hours of processing time and additionally the reduction of deviations in the results due to manual labor.

## 1 Introduction

Aneurysmal subarachnoid hemorrhage (aSAH) has a worldwide incidence of 8 per 100 000 person-years and carries an exceptionally high disease-specific burden in terms of mortality and long-term disability (1, 2). aSAH is due to rupture of an aneurysm, resulting in bleeding into the cerebrospinal fluid (CSF) filled subarachnoid space (3). Brain injury from aSAH occurs in an early and delayed phase (3). Early brain injury is caused by increasing intracranial pressure, decreased cerebral blood flow, transient global ischemia and early toxic effects of subarachnoid blood (4). Uniquely in aSAH, a delayed phase of brain injury due to delayed cerebral ischemia (DCI) follows in a third of patients 3–14 days after the initial bleeding (3). DCI leads to secondary infarctions and is the most important cause of long-term disability in patients surviving the initial hemorrhage (5). After erythrocytolysis in the subarachnoid CSF space, cell free hemoglobin (CSF-Hb) can be detected as oxygenated hemoglobin (oxyHb) by spectrophotometry. CSF-Hb contributes critically to the development of DCI (6, 7). Previously, we found that CSF-Hb strongly interferes with vascular nitric oxide signaling of isolated cerebral arteries (8). OxyHb infused in the CSF of sheep induces highly reproducible acute vasospasms.

Acute hydrocephalus occurs in 20% of patients with aSAH (9) and is treated with a CSF diversion device (external ventricular drain or lumbar drain) in the acute stage. CSF is drained regularly and collected in measuring containers and is normally discarded. This provides the unique opportunity in aSAH patients for regular CSF analyses without additional invasive procedures.

Continuous bedside measurement of free CSF-Hb in aSAH would allow to identify the patient-specific time-profile of erythrocytolysis in the subarachnoid space and to monitor for the risk of DCI at every time point of disease. These measurements guide new therapeutic interventions to reduce CSF-Hb toxicity in aSAH patients (6). So far, repetitive CSF spectrophotometry to monitor CSF-Hb levels after aSAH in patients is a manual process and reserved for highly specialized centers. Today, no point-of-care diagnostic test (POCT) system exists that allows continuous and standardized testing. CSF is sampled from the external ventricular drains, centrifuged to exclude intact erythrocytes and then CSF-Hb spectrophotometrically quantified in the supernatant. This approach is prone to in-vitro hemolysis if the sample is not processed immediately. Erythrocytes release their hemoglobin before centrifugation and possibly also during centrifugation, which leads to falsely high measured values for cell-free Hb. Such an effect can be mitigated by a POCT system with standardized and immediate sample processing. A study by Lenshof et al. (10) presented a microfluidic device that allows to extract clinical grade plasma from whole blood samples by acoustic forces, which are gentle and allow the cells a high viability. Petterson et al. (11) used this principle to determine hematocrit. This study presents a chip based on the design of Lenshof et al. that can serve as the basis for a POCT system for continuous monitoring of CSF-Hb levels in aSAH patients without the need for sample centrifugation.

## 2 Materials and methods

### 2.1 Plasmapheresis chip

**Figure 1:**
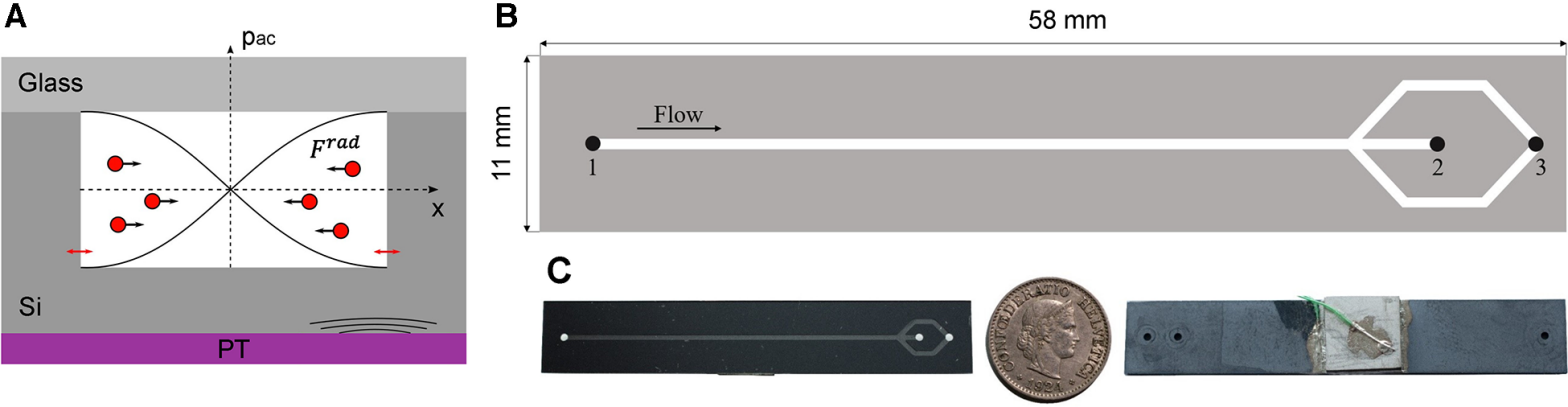
(**A**) Shematic of the device cross section illustrating the working principle used for the separation of erytrocytes from CSF or blood plasma. The piezoelectric transducer (PT) causes nm-displacements on the cavity walls (red arrows) inducing an acoustic pressure in the microchannel (p_ac_). By generating a standing wave in the microchannel, the erythrocytes are forced by F^rad^ to the acoustic pressure node in the centre of the channel. (**B**) Top view of the plasmapheresis chip. Inlet (1) of the mixture of CSF and blood sample. The focused erythrocytes are collected at the waste outlet (2). The CSF-Hb or blood plasma is not affacted by the acoustic radiation force and can thus be extracted at the corresponding outlet hole (3). **C** Illustration of the size of the actual chip by a 5 cent Swiss Franc coin.

A prototype of a microfluidic chip was produced that is capable of separating erythrocytes from CSF-Hb using acoustic forces based on the design by Lenshof et al. (10) Manipulation of cells using acoustic forces (acoustophoresis) is based on the generation of an acoustic pressure in a microfluidic channel via a piezoelectric element. At a defined frequency, an acoustic standing wave is generated in the channel with the pressure node in the center of the channel (12). The scattering of the acoustic waves at the cells generates a force called the acoustic radiation force (F^rad^) pushing them towards the acoustic pressure node (Figure 1A) (12–14). The erythrocytes are focused in the center of the channel due to their positive acoustic contrast factor with respect to blood plasma and CSF (see Supplementary Material).

The device is composed of a base made of silicon and was produced by double-sided photolithography on a silicon wafer with 4-inch diameter and 500 µm ± 10 µm thickness (Figure 1B). The microfluidic channel and access holes were produced by inductively coupled plasma deep reactive ion etching (ICP-DRIE, Bosch process, Estrellas, Oxford instruments) on the front and back side with a channel depth of 200 µm ± 5 µm, a channel width of 700 ± 5 µm, and access holes diameter of 1,000 µm.

**Figure 2:**
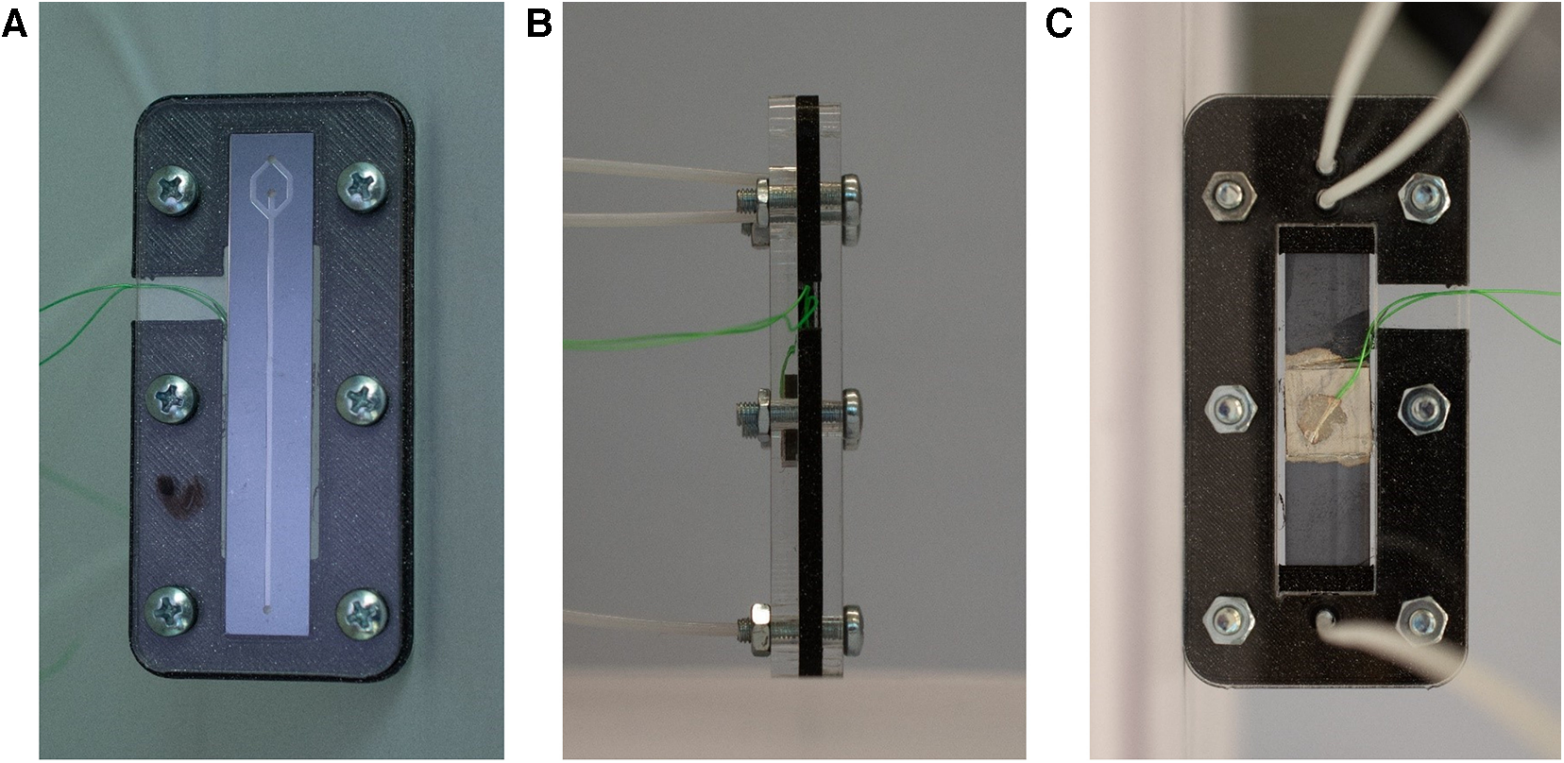
Illustration of the holder for the microfluidic chip. With a clamping system, the tubes are connected to the respective holes in the chip. Additionally, the cables are accesible to actuate the piezoelectric element. (**A**) Top view of the chip clamped in the holder. (**B**) Side view. (**C**) Bottom view with the piezoelectric transducer and the tubes connected to the inlet and outlet holes.

A glass wafer (thickness 700 µm) was anodically bonded (SB6, SÜSS MicroTec, Garching, Germany) to the silicon wafer and then diced with a wafer saw (DAD3221, Disco corporation) into rectangular chips (11 mm × 58 mm). The piezoelectric transducer (20 mm × 20 mm × 2 mm, PZ26, Ferroperm Piezoceramics A/S, Kvistgard, Denmark) was coupled to the backside of the diced chips with an epoxy resin (H20E, EPO-TEK). Two copper wires (0.25 mm diameter) were glued to the electrodes of the piezoelectric transducer using silver paste and instant glue. The chip was clamped in a holder and sealed with O-rings with three flanged PTFE tubes (1/16-inch × 0.75 mm outer and inner diameter, respectively), which allow to access the inlet and the two outlet holes (Figure 2).

### 2.2 *In vitro* test setup

**Figure 3:**
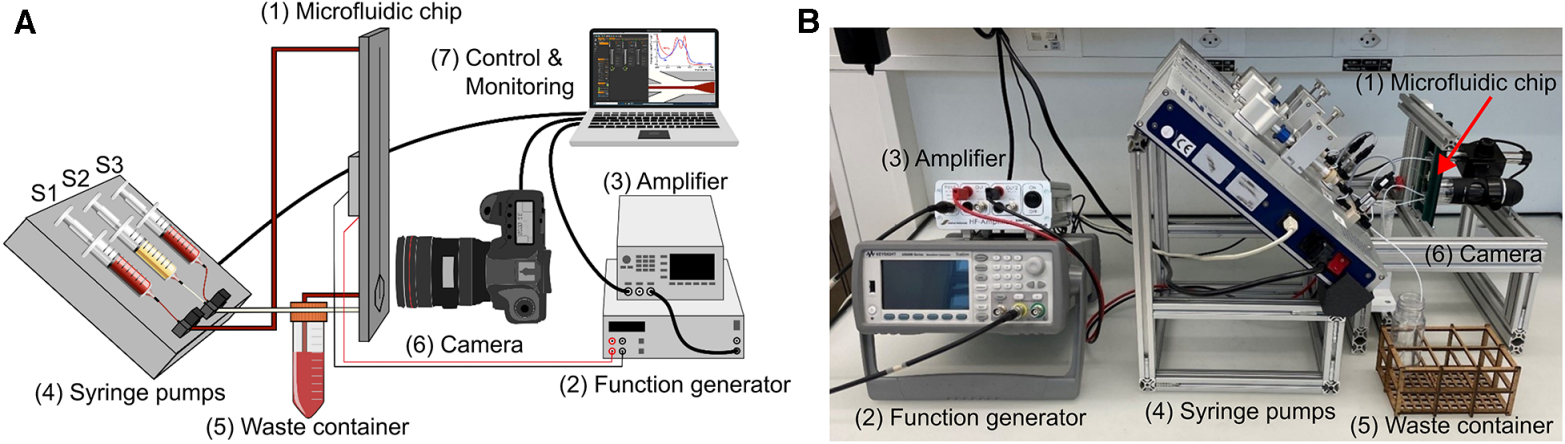
Illustration of the *in vitro* test bench. It consists of the microfluidic chip (1), the function generator (2) and amplifier (3), the three syringe pumps (4) with Syringe 1 (S1) connected to the inlet of the chip and to the sample vessel, Syringe 2 (S2) connected to the plasma outlet of the chip and Syringe 3 (S3) to the waist outlet, the waste container (5) connected to the center outlet, an optical evaluation (6), and a PC for control and monitoring (7). (**A**) schematic illustration of the test bench. (**B**) Test bench in the laboratory setting.

An *in vitro* test bench was built (Figure 3), structured in a modular way, to test the microfluidic chip. It consists of an interface to attach the particle separation device and the spectrophotometer, three syringe pumps (Nemesys 290N, low pressure syringe pump, Cetoni GmbH, Korbussen, Germany) and tubing with 0.75 mm diameter. The electronics to operate the piezoelectric transducer consist of a waveform generator (333500B Series, Keysight, CA, USA) and a High Wave 3.2 amplifier (Digitum Elektronik, Nürtingen, Germany). A camera (Toolcraft DigiMicro, Conrad Electronic, Hirschau, Germany) was used for imaging at the bifurcation of the microfluidic chip. In addition, the extended test environment included a laboratory spectrometer (Photometer 4040v5+, Riele, Berlin, Germany) and a centrifuge (Universal 320 & Hematocrit Rotor, Hettich AG, Bäch, Switzerland) to test the same samples in parallel. This allows us to evaluate and compare each test of the plasmapheresis chip with the standard analysis.

### 2.3 Testing procedure

**Figure 4:**
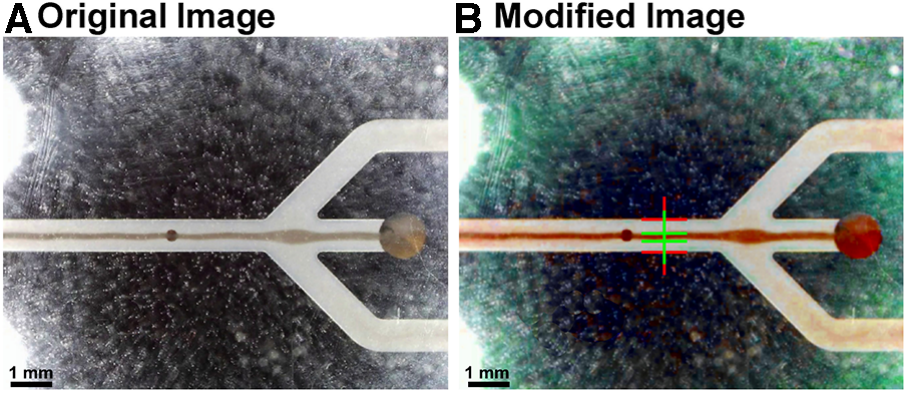
Images depicting the identification of the optimal operating point. (**A**) The original image was modified with a MATLAB script. It enhances the image contrast and allows to (**B**) automatically detect the line width of the acoustically focused red blood cells, hence, the focusing of the particles. The input voltage of the piezoelectric transducer was continuously increased and the ratio between the channel and the line width of the cells measured (red and green lines, respectively).

The microchips were tested with diluted porcine blood and CSF samples from six aSAH patients. The porcine samples were diluted with phosphate buffered saline (PBS) to mimic the same hematocrit level as for the CSF case, whereas the patient samples were not diluted. In order to remove any air inclusions, the entire system, i.e., the tubes and the microfluidic chip, are flushed with the sample to be tested and collected at a waste container. In detail, the first syringe (S1) is connected via the first three-way valve to the inlet of the chip and to a vessel containing the sample, which is used for filling S1. The waste outlet of the chip is connected to a waste collection container and the plasma outlet is connected to the second three-way valve to which the second syringe (S2) and the third syringe (S3), the waste syringe, are connected (Figure 3). All syringes are positioned at an angle. After initial flushing of the system the flow rate at the inlet is set to 60 µL/min and to 15 µL/min at the plasma outlet (Figure 1, outlet No. 3). At these flow rates, the device could be operated for hours without any adjustment of the frequency, voltage, and flow rate while still avoiding any contamination of red blood cells in the plasma outlet. The flow rates at inlet and outlet could be increased leading to higher throughput and amount of plasma extracted. However, an increased flow rate could lead to contamination of the plasma outlet due to small variations during operation such as temperature. Therefore, we utilized more conservative parameters to allow small deviations during operation leaving the purity of the plasma outlet unaffected. At steady state flow, the piezoelectric transducer is actuated causing the erythrocytes to move towards the center of the channel (Figure 4). The input signal to operate the piezoelectric transducer is a sinusoidal wave with a frequency range of 1,036–1,050 kHz [half wavelength fitted into the channel width (*λ*/2-mode), Figure 1A, detailed in Supplementary Material] and a peak-to-peak voltage of 28 V, keeping the operating temperature always below 30 °C.

The optimal operating point for the active blood particle separation device is evaluated by applying different voltages and frequencies on the piezoelectric transducer as well as different flow rates at the inlet and outlet. The resulting efficiency of focusing the blood particles are monitored (Figure 4). The separation of blood particles is shown in the Supplementary Movie (OxyHbMeter_FunctionalPrinciple). Note, in the Supplementary Movie, the fluid flows from left to right and is separated as follows: waste (separated red blood cells) being extracted in the middle and plasma or CSF for CSF-Hb detection is located in the two side arms.

After all the dead volume in the tubes is drained out, the plasma or CSF is collected in the corresponding syringe. The operation of the system is continued for about 70 min until 1 mL of plasma or CSF is collected in the plasma or CSF syringe. The collected plasma or CSF is then analyzed with the spectrophotometer to assess the Hb concentration of the sample. Afterwards the device was cleaned with saline solution and ethanol, ready to be reused for the next experiment.

### 2.4 Clinical setup

In the clinical setting, CSF samples from six patients treated at the Neurocritical Care Unit University Hospital Zurich with aSAH and hydrocephalus with need for an external ventricular drain were analyzed. The study was approved by the local ethics committee of the canton of Zurich, Switzerland, Approval Nr. 2021-01089. Written informed consent was given by legal representatives, as all patients were incapable of judgment and the study was conducted in accordance with the declaration of Helsinki and its amendments. CSF was taken from the collection bag containing the CSF drained over the past 2–3 h.

**Figure 5:**
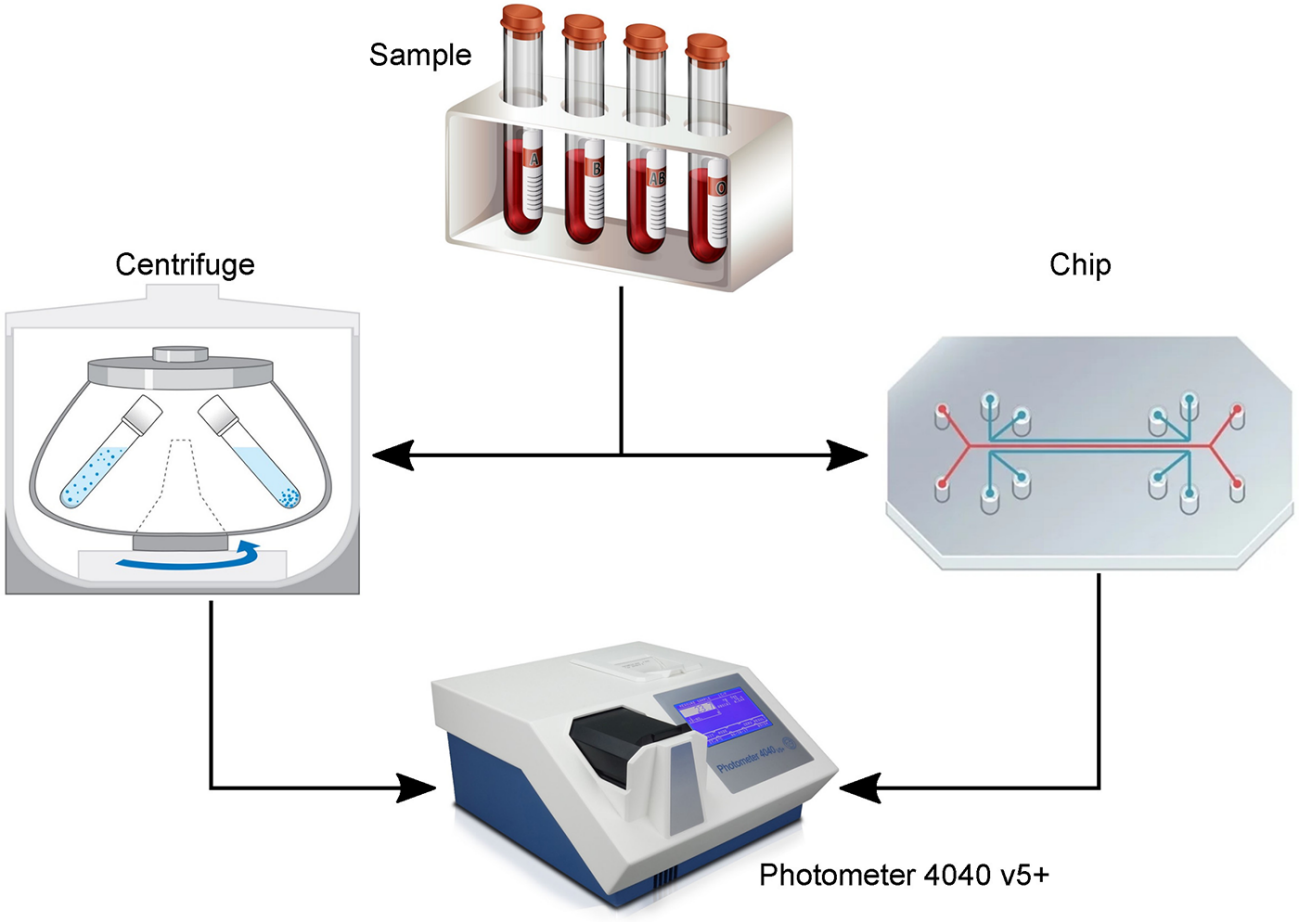
Illustration of the experiments to evaluate the performance of the plasmapheresis chip. The sample [diluted blood or patient cerebrospinal fluid (CSF)] was either centrifuged or separated with the chip and the remaining plasma or CSF measured with a laboratory photometer for their hemoglobin concentration.

The separation efficiency and the hemolysis induced by the separation of the microfluidic chip were compared to conventional centrifugation (Figure 5). The samples were centrifuged at 3,600 RCF for 15 min and the separated CSF was analyzed spectrophotometrically. The measured Hb values from the centrifuged sample served as a baseline to verify the values from the separated sample with the microfluidic chip. For the spectrometer analysis of both separation methods, five cuvette measurements were performed.

## 3 Results

With the most recent chip design (Figure 1) and test setup (Figure 3), *in vitro* and clinical measurements were made with diluted blood and CSF patient samples, respectively. These experiments served to evaluate the separation efficiency and the hemolysis induced by the separation of the microfluidic chip compared to conventional centrifugation (Figure 5).

### 3.1 *In vitro* testing

Table 1 shows the results of the measurements performed with porcine blood. The blood was diluted with PBS to a hematocrit (HCT) value of 10% and then separated with the microfluidic chip. In this experiment, samples separated with the microfluidic chip showed increased Hb concentration compared to samples after centrifugation. Due to the high HCT in the sample and the geometric constraints, not all cells could be efficiently separated, leaving blood cells in the extracted plasma. Therefore, the Hb within the remaining blood cells confounded Hb quantification. Despite the slight overestimation of the hemoglobin concentration, the results of the plasmapheresis chip were very close to those obtained by centrifugation, which was a promising result justifying the testing with patient samples.

**Table 1:** Comparison of the centrifugation and the plasmapheresis chip of the measurements with porcine samples.

#	Centrifugation		Plasmapheresis chip		Difference
	Mean (mg/dL)	SD (mg/dL)	Mean (mg/dL)	SD (mg/dL)	(%)
1	1.786	±0.157	2.068	±0.152	14.6
2	2.349	±0.035	3.358	±0.291	35.4
3	0.899	±0.043	0.970	±0.089	7.6
4	6.466	±0.295	7.513	±0.199	15.0

The samples were diluted with PBS to a HCT of 10%. The results show the measured hemoglobin concentration in the extracted plasma in mg/dL. Data is listed as mean, standard deviation (SD) as well as relative differences (|x1-x2|/(x1 + x2)/2)*100) between the two measurement principles.

### 3.2 Clinical testing

**Figure 6:**
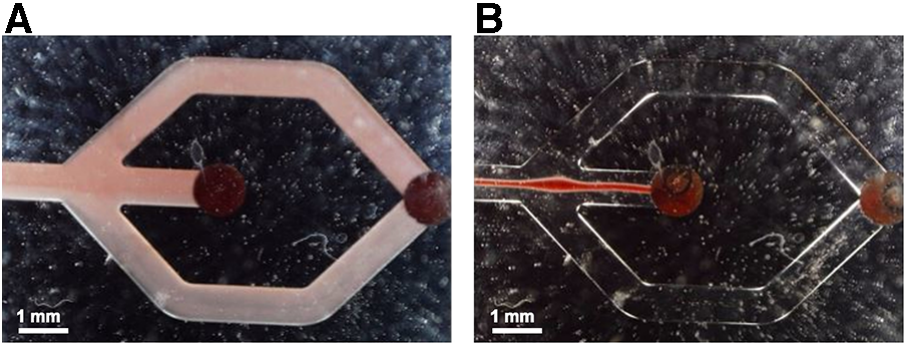
Flow observed in the microfluidic channels without (**A**) and with (**B**) acoustic actuation, respectively. In B the acoustic forces lead to a narrow band of erytrocites in the channel centre with no visible spillover to the side outlets at the chosen operation conditions.

In a next step, we tested our plasmapheresis chip with CSF samples obtained from the external drain of patients that have a brain injury from aSAH. As mentioned before, there are only a few people per year that attain such an injury, hence the low number of data points. In Figure 6 measurements with patient sample No. 1 (Table 2) are shown that had a hematocrit level of 5%. When turning on the piezoelectric element and hitting the resonance frequency of the channel, the erytrocites are focused in a small band in the centre of the channel and thus exit only through the central outlet (Figure 1, outlet Nr. 2).

**Table 2:** Comparison of the centrifugation and the plasmapheresis chip of the ex vivo measurements with patient samples.

#	Centrifugation		Plasmapheresis Chip		Difference
	Mean (mg/dL)	SD (mg/dL)	Mean (mg/dL)	SD (mg/dL)	(%)
1	0.72	±0.035	0.54	±0.232	28.6
2	2.36	±0.062	2.55	±0.101	7.7
3	58.39	±1.368	59.90	±0.252	2.6
4	48.52	±0.715	48.13	±0.551	0.8
5.1	1.82	±0.037	1.80	±0.072	1.1
5.2	1.81	±0.035	1.85	±0.028	2.2
6.1	1.69	±0.022	1.72	±0.037	1.8
6.2	1.59	±0.012	1.63	±0.032	2.5

The results show the measured hemoglobin concentration in the extracted CSF mixture. Data is depicted as mean, standard deviation (SD) as well as the relative differences (|x1-x2|/(x1 + x2)/2)*100) between the two measurement principles. The first column indicates samples from different patients, where 5.1 and 5.2 as well as 6.1 and 6.2 are individual measurements from the same patient samples.

**Figure 7:**
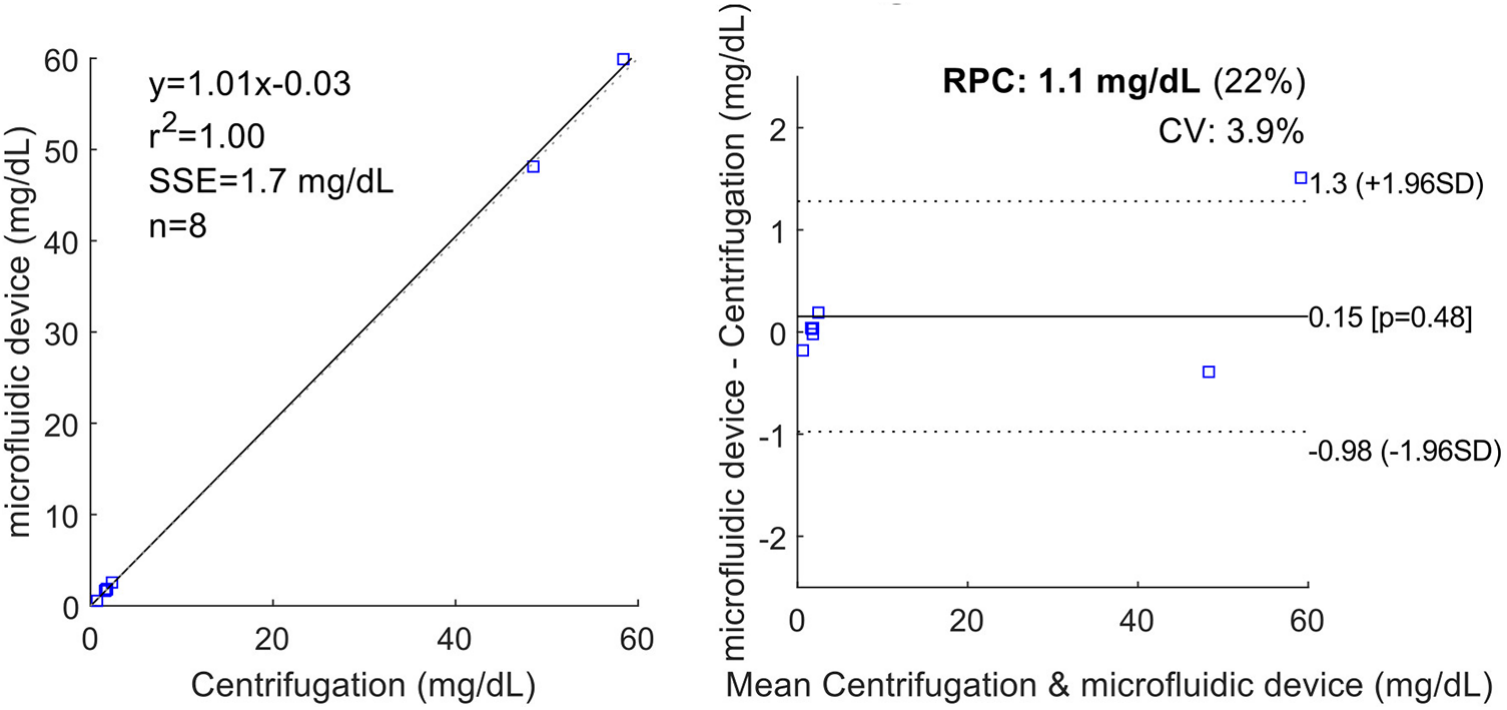
Correlation and Bland-Altmann plot of the patient samples (Table 2) (15). The plot demonstrates high correlation between the two methods with slightly higher inaccuracy towards high oxygen concentrations.

With the most recent chip design and test setup, ex vivo measurements were made with samples from six patients. Table 2 lists the results of the measurements performed. All patient samples had a HCT of <5% used for the performance tests. The results show that the measured Hb concentrations in the extracted CSF (centrifugation and microfluidic chip) are in a similar range for the patient samples. The minor differences in these results can be attributed to handling errors such as pipetting. The good agreement of the two methods is further supported by a correlation and Bland-Altmann plot (Figure 7).

## 4 Discussion

The novel bedside monitoring system should allow for continuous and noninvasive quantification of CSF-Hb, without the need for disconnection or puncturing of the CSF drainage system. Compared to daily CSF sampling with manual processing of each sample, the OxyHbMeter would offer increased usability, reduce the risk for infection and provide an autonomous measurement of CSF-Hb concentrations. This method could improve reproducibility and be integrated in an effective warning system by providing a biomarker to predict DCI (6, 7). With the current study, we have shown proof of principle with comparable results to centrifugation.

During testing, the flow rates were heuristically chosen to have stable focusing of the particles throughout the extraction of plasma or CSF. The chip used for this study had an optimal input flow rate of 60 µL/min at the inlet and 15 µL/min at the CSF outlet, balancing the concentration of the particles and the yield at the plasma and CSF outlet guaranteeing stable operation for hours, meaning that the experimental parameters (voltage, frequency, and flow rate) didn't have to be adjusted. The effective flow rate was constraint mainly by the length of the channel and the HCT value of the sample. Moreover, the focusing strength in the channel could be controlled by the applied voltage on the piezoelectric transducer, however, a higher voltage resulted in an increased temperature of the device harming the cells. In this study, we applied a peak-to-peak voltage of 28 V, keeping the operating temperature always below 30 °C. Generally, by increasing the length of the channel or increasing the acoustic energy density in the channel by side actuation (16), or active cooling (17) higher flow rates would be possible to increase the throughput and thus a higher yield at the CSF outlet in clinical scenarios. In the literature, flow rates of up to 400 µL/min have been demonstrated for a similar setup and application (18). Even at these high flow rates, detrimental effects such as cell lysis have not been observed. Further increasing the flow rate to >600 µL/min leads to hydrodynamic phenomena that need to be considered (19). The limitation in throughput is one of the main reasons why acoustofluidic devices have yet very rarely found the way to clinical applications.

The test setup was designed with a 45° inclination of the syringe pumps. With this the sedimentation of the erythrocytes to the bottom of the syringes could be delayed, allowing for a stable HTC in the sample throughout the experiment. The chip stability is of enormous importance. Air bubbles or temperature dependent optimal frequency changes were confounders that were repeatedly detected during the measurements, which meant that the measurements had to be interrupted or restarted. In future experiments, the device could not only be cleaned but prefilled with ethanol to push out all air that was remaining in the system. Furthermore, the frequency shift due to temperature could be handled by a frequency tracking system as presented by Hammarström et al. (20).

Processing samples with the microfluidic device in our *in vitro* setup took longer than centrifugation. However, in the future, it may result in more standardized CSF-Hb measurements with prompt and autonomous processing of CSF. Samples are not waiting to be sent to the laboratory for unspecified times nor are they exposed to different centrifugation speeds. After separating CSF with the microfluidic device, the measured CSF-Hb-values were in a similar range compared to measurements after centrifugation. There was hardly any additional hemolysis induced by the device. However, a major limitation of this study is the limited available number of clinical samples from aSAH patients. Further, the implementation of the microfluidic device into the draining system has yet to be demonstrated. However, the performance of the current device is good enough to justify further investigation into this direction.

In our test setting, a low HCT within the sample was beneficial over a higher one as increased viscosity tended to occlude the channel and made separation more prone to errors. Residual contaminations within the system could be an additional source for measurement differences, even though the chip and all tubes were thoroughly cleaned before each experiment with diluted blood and a new chip was used for the samples from each patient.

## 5 Conclusion and outlook

The preliminary tests with the plasmapheresis chip show that it is capable to continuously separate erythrocytes from diluted blood and CSF samples, enabling standardized in-line testing of biomarkers. There is no additional hemolysis induced by the microfluidic chip compared to centrifugation, and the performance is comparable. The developed platform serves as a solid base for the future development of a point of care device.

Further, we envision a platform technology suitable for several applications: First, in aSAH the continuous measurement of CSF-Hb could advance our understanding of DCI, biosignal analytics and cognitive computing, first in research and in a second step in its timely treatment (8). Data would be processed in real time and results presented as a DCI prediction, prevention and decision support system. Deep phenotyping and advanced biosignal analytics, furthermore, will contribute to a profound understanding of the complex pathophysiology of DCI after aSAH. CSF-Hb, as a biomarker for DCI, may allow to identify high risk patients as a target population to study new drugs, e.g., the hemoglobin-scavenger haptoglobin (6, 8). Second, computed tomography (CT) is the diagnostic test of choice to diagnose subarachnoid hemorrhage in patients with acute headache. Failure to obtain a timely CT scan and its low sensitivity a few days after headache onset may lead to missed diagnosis (3). Lumbar puncture allows to diagnose subarachnoid hemorrhage if xanthochromia is present in the supernatant from centrifugated CSF samples. Xanthochromia is used to distinguish the elevated red blood cell count (RBC) observed in the CSF of SAH from the elevated RBC count observed after traumatic lumbar puncture and is considered present if the absorption follows a characteristic oxyhemoglobin curve (21). False positive test results can only be avoided if the CSF is centrifugated and spectrophotometrically analyzed immediately. A procedure which is reserved for highly specialized lab units because analytical methods require certified technical personnel. A POCT system might allow for 24/7 CSF spectrometry in the environment of an Emergency or Intensive Care Unit. And third, not only the assessment of CSF-Hb in a more frequent and standard manner could benefit from the separation principle (12), but also other CSF analytics, such as cytology. The cellular composition of the CSF provides important first information across a broad spectrum of inflammatory central nervous system diseases. For cytometry, CSF samples require immediate centrifugation and analysis, ideally within 1 h from collection (22).

## Data Availability

The raw data supporting the conclusions of this article will be made available by the authors, without undue reservation.
