# Supplementary material for: OxyHbMeter—a novel bedside medical device for monitoring cell-free hemoglobin in the cerebrospinal fluid—proof of principle
Source: Front Med Technol. 2024 Apr 11;6:1274058. doi: 10.3389/fmedt.2024.1274058 (PMC11043468; doi:10.3389/fmedt.2024.1274058)
Supplement: Supplementary file 2 [file Datasheet1.pdf]

Supplementary Material

OxyHbMeter – a novel bedside medical device for monitoring cell-free hemoglobin in the cerebrospinal fluid – proof of principle

Nikolaos Tachatos[1†], Jan Folkard Willms[2†], Michael Sebastian Gerlt[3], Kiran Kuruvithadam[1], Michael Hugelshofer[4], Kevin Akeret[4], Jeremy Deuel[5], Emanuela Keller[2], Marianne Schmid Daners[6]

[1]Product Development Group Zurich, Department of Mechanical and Process Engineering, ETH Zurich, Zurich, Switzerland

[2]Neurointensive Care Unit, Department of Neurosurgery and Institute of Intensive Care Medicine, Clinical Neuroscience Center, University Hospital Zurich and University of Zurich, Zurich, Switzerland

[3]Biomedical Engineering Department, Lund University, Lund, Sweden

[4]Department of Neurosurgery, Clinical Neuroscience Center, University Hospital Zurich and University of Zurich, Zurich, Switzerland

[5]Department of Medical Oncology and Haematology Clinic, Universitätsspital und University of Zurich, Zurich, Switzerland

[6]Institute for Dynamic Systems and Control, Department of Mechanical and Process Engineering, ETH Zurich, Zurich, Switzerland

[†]These authors contributed equally to this work and share first authorship.

*** Correspondence:**

Marianne Schmid Daners
marischm@ethz.ch

Key Terms

Delayed cerebral ischemia, aneurysmal subarachnoid hemorrhage, microfluidics, acoustic particle manipulation, cell separation, point of care device, centrifugation, spectrophotometry

Supplementary data

1 Materials and methods

1.1 Acoustophoresis – governing equations

The principle of acoustophoresis is based on the generation of an acoustic pressure in a microfluidic channel via a piezoelectric element device (Figure 1). The acoustic pressure generates an acoustic radiation force on the particles. At a defined frequency, a standing wave is generated in the channel with the node in the center of the channel. The resonance frequency (f) depends on the channel geometry and fluid properties. The wavelength (λ) is derived from the channel width (D) and the chosen resonance mode (k). To focus the particles in the middle of the channel along the channel width (needed for our application of geometric separation), the first mode is used where half of the wavelength fits into the channel width (k = 1, λ/2-mode). The frequency is dependent on the wavelength and the speed of sound (c_0) in the used fluid:

$$f = \frac{c_0}{\lambda} \quad \text{with} \quad \lambda = 2Dk \tag{1}$$

Using these formula, we can approximate the resonance frequency of our device. Our channel has a width of D = 700 µm, we are interested in focusing particles in the center hence k = 1, leading to a wavelength of λ = 1.4 mm. Considering the speed of sound in water c_0 = 1500 m/s results in a frequency of f = 1.07 MHz which is very close to the frequency we used in our experiments.

In a straight and rectangular micro-channel environment, the acoustic wave can be approximated as a standing, resonant pressure wave of the following form

$$p_{ac} = p_a \cos(kz)\sin(2\pi ft) \tag{2}$$

The acoustic radiation force is the time-averaged force that acts on particles suspended in a fluid which are exposed to an acoustic standing wave field. The force results from differences between the particle and the surrounding fluid, such as the compressibility (κ), the density (ρ) of the fluid and the particle, the size of the particle as well as the frequency and the acoustic energy density of the acoustic standing wave.

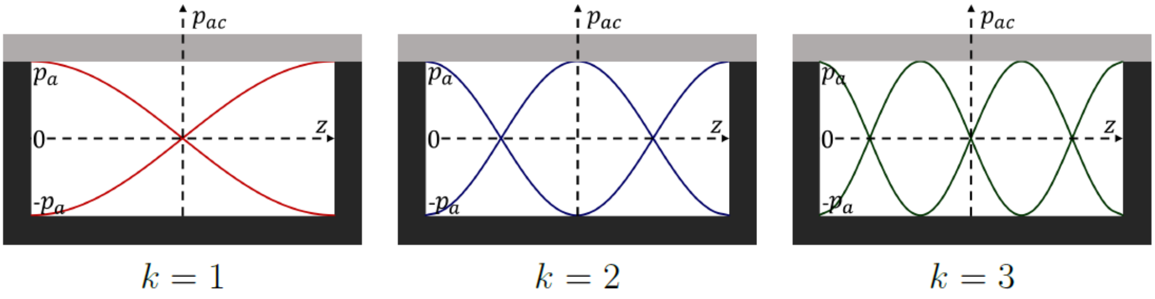

Supplementary Figure 1 Sketches of transverse, standing plane and ultrasound waves within a straight, hard-walled and fluid filled channel with different resonant modes (k = 1, 2 and 3, respectively) (2).

As per Gor'kov (1), the acoustic radiation potential, alias Gorkov Potential, (U^{rad}) is defined as:

$$U^{rad} = 2\pi r_p^3 \left(f_1 \frac{1}{3\rho_0 c_0^2}\langle p_{in}^2\rangle - f_2 \frac{\rho_0}{2}\langle v_{in}^2\rangle\right) \tag{3}$$

$$f_1 = 1 - \tilde{\kappa} \quad \text{with} \quad \tilde{\kappa} = \frac{\kappa_p}{\kappa_0} = \frac{\rho_0 c_0^2}{\rho_p c_p^2} \tag{4}$$

$$f_2 = \frac{2(\tilde{\rho} - 1)}{2\tilde{\rho} + 1} \quad \text{with} \quad \tilde{\rho} = \frac{\rho_p}{\rho_0} = 2\left(\frac{\rho_p - \rho_0}{2\rho_p - \rho_0}\right) \tag{5}$$

r_p denotes the radius of the particle, while ρ_p is the density, κ_p and c_p the speed of sound of the particle. ρ_0, κ_0 and c_0 denote the density, the compressibility, and the speed of sound of the fluid respectively. $\tilde{\rho}$ and $\tilde{\kappa}$ stand for the relative density and compressibility, while $\langle p_{in}^2\rangle$ and $\langle v_{in}^2\rangle$ stand for the time-averaged incident pressure and velocity squared, respectively. f_1 is obtained from the mass flow related to the compressibility, while f_2 is determined from the translational motion. Based on the

above terms and equations the formula for the acoustic radiation force (F^{rad}) acting on a particle towards the acoustic pressure nodes of the standing wave field is derived by taking the negative gradient of the Gor'kov Potential:

$$F^{rad} = -\nabla U^{rad} = -\frac{4\pi}{3} r_p^3 \nabla \left(f_1 \frac{1}{2\rho_0 c^2} \langle p_{in}^2 \rangle - f_2 \frac{3}{4} \rho_0 \langle v_{in}^2 \rangle \right) \qquad 6)$$

The magnitude and direction are dependent on the acoustic energy density E_{ac} and the acoustic contrast factor ϕ, respectively - for our fluid and the particle, it is expressed as:

$$E_{ac} = \frac{p_a^2}{4\rho_0 c_0^2} = \frac{1}{4} \rho_0 v_a^2 \qquad 7)$$

$$\phi = \frac{1}{3} f_1 + \frac{1}{2} f_2 = \frac{1}{3} \left(\frac{5\tilde{\rho} - 2}{2\tilde{\rho} + 2} - \tilde{\kappa} \right) \qquad 8)$$

In this equation p_a represents the pressure and v_a the velocity amplitude. The contrast factor can result in a negative number. If that is the case the particles are forced towards the pressure anti-nodes and away from the pressure nodes. In our application, the blood cells possess a positive acoustic contrast factor with respect to the fluid they are dissolved in i.e., the blood plasma and CSF, enabling us to focus them in the pressure node and thus separating them (3).

Bibliography

1. Gor'kov, L. P. On the Forces Acting on a Small Particle in an Acoustical Field in an Ideal Fluid. Soviet Physics Doklady 6:773, 1962.
2. Laurell, T., F. Petersson, and A. Nilsson. Chip integrated strategies for acoustic separation and manipulation of cells and particles. Chemical Society reviews 36:492–506, 2007.
3. Lenshof, A., A. Ahmad-Tajudin, K. Järås, A.-M. Swärd-Nilsson, L. Aberg, G. Marko-Varga, J. Malm, H. Lilja, and T. Laurell. Acoustic whole blood plasmapheresis chip for prostate specific antigen microarray diagnostics. Analytical chemistry 81:6030–6037, 2009.
